# SNPoptimizer: a scalable genetic-algorithm framework to derive minimal discriminatory SNP panels from large genotyping datasets

**DOI:** 10.1007/s11032-026-01707-z

**Published:** 2026-08-29

**Authors:** Salvatore Esposito, Nicola Scalzi, Samuela Palombieri, Walter Sanseverino, Francesco Sestili, Alessandra Stella, Raffaella Balestrini, Stefania Grillo, Ray Anthony Bressan, Giorgia Batelli

**Affiliations:** 1 Institute of Biosciences and Bioresources Research Division Portici, National Research Council of Italy (CNR-IBBR), Via Università, 133, Portici, Italy; 2 Department of Agriculture and Forest Sciences (DAFNE), University of Tuscia, Via S. Camillo de Lellis, Viterbo, Italy; 3 Valuegroovers srl, Via S. Camillo de Lellis, Viterbo, Italy; 4 Sequentia Biotech, Carrer de València, Barcelona, SL Spain; 5 Institute of Agricultural Biology and Biotechnology, National Research Council of Italy (CNR-IBBA), Via Alfonso Corti, 12, Milan, Italy; 6 Institute of Biosciences and Bioresources, National Research Council of Italy (CNR-IBBR), Via Amendola 165/A, Bari, Italy; 7 West Lafayette, IN USA

**Keywords:** SNP selection, Genotype discrimination, Shiny app, Population genomics, Precision breeding

## Abstract

The ability to efficiently discriminate genotypes is a critical step in genomics-assisted breeding, population genomics, biodiversity studies, traceability along food chains, and germplasm management. However, identifying the minimal and most informative subset of SNPs capable of uniquely distinguishing a large set of individuals remains a computationally challenging task. Here, we present SNPoptimizer, a user-friendly Shiny application that uses a genetic algorithm–based framework to optimally select discriminatory SNPs from large-scale genotyping datasets. By leveraging the evolutionary principles of selection, mutation, and crossover, SNPoptimizer iteratively identifies compact SNP panels that maximize genotype resolution. The application supports HapMap-formatted and VCF genotype files and includes an optional second-round optimization for resolving putative duplicates. We benchmarked SNPoptimizer across three independent datasets, including a tomato diversity panel, 820 Cauliflower genotypes, and a soybean diversity panel comprising 30 million variants across 1,511 samples. Across the three datasets, panels of 17–22 SNPs yielded R-VDP values ranging from 0.8744 to 0.9973, with complete discrimination obtained in Dataset III, demonstrating robust performance across different datasets. Cross-tool comparisons revealed complementary trade-offs among discriminatory power, panel size, runtime, and run-to-run reliability. SNPoptimizer provides a flexible solution for researchers seeking to reduce genotyping costs while maintaining high discriminative power.

**Supplementary Information:**

The online version contains supplementary material available at https://doi.org/10.1007/s11032-026-01707-z.

## Introduction

Accurate identification and discrimination of plant genotypes are fundamental to germplasm management, variety registration, and transparent value chain traceability. Mislabeling or redundancy among accessions in gene banks or breeding programs can lead to inefficient conservation and use of resources, particularly in vegetatively propagated or clonally maintained species. To ensure trueness-to-type and genetic integrity, molecular fingerprinting has become an essential complement to phenotypic evaluation (Zhang et al. 2022; Eissa et al. 2023; Yuan et al. 2025).

Varietal identification based on genetic fingerprinting relied on microsatellite (SSR) markers (Garkava-Gustavsson et al. 2008; Evans et al. 2011), appreciated for their multi-allelic and co-dominant nature but limited by low throughput, manual scoring, and reproducibility issues. With the advent of next-generation sequencing (NGS), high-density Single Nucleotide Polymorphism (SNP) arrays have largely replaced SSRs, providing reproducible and scalable platforms for genome-wide genotyping in several crop species, including wheat, barley, potato, tomato, and apple (Allen et al. 2017; Bayer et al. 2017; Vos et al. 2015). Although these platforms facilitate powerful genomic studies and enable genomic selection applications, their implementation remains costly and often impractical for routine identification or large-scale screening of germplasm collections, where only a small set of highly informative markers is required. Current strategies for SNP subset selection generally rely on summary statistics such as minor allele frequency (MAF), heterozygosity, polymorphism information content (PIC), or other genetic indices (Mata-Nicolás et al. 2020; Winfield et al. 2020; Hoon et al. 2023; Sales et al. 2023). While these metrics are useful for assessing diversity, they are not specifically designed to optimize genotype-level resolution. Consequently, the performance of these methods may be suboptimal when the objective is the complete discrimination of all genotypes in a dataset using a minimal number of markers (e.g. the case of rare/unique alleles/haplotype).

In recent years, the increasing complexity and scale of genotypic datasets have led to the adoption of data-driven and artificial intelligence (AI)-based approaches in genomic research. Among these, machine learning and evolutionary algorithms (EA) have shown great promise for solving high-dimensional optimization problems (Molina et al. 2025; Shao et al. 2025). In marker selection, the number of possible SNP combinations grows exponentially with the number of loci, making exhaustive search computationally intractable even for moderately sized datasets. Evolutionary algorithms, including genetic algorithms (GA), are particularly suited to this challenge, as they efficiently explore large and complex search spaces by mimicking natural selection processes through iterative selection, recombination, and mutation of candidate solutions, enabling the identification of near-optimal subsets of markers that maximize a given fitness function.

Building on this conceptual framework, we developed SNPoptimizer, a Shiny-based R application that applies a genetic algorithm to identify sets of SNPs required to achieve unique genotype discrimination within any given dataset. Unlike frequency-based methods (e.g., based on minor allele frequency, polymorphism information content, or heterozygosity; Winfield et al. 2020; Hoon et al. 2023; Sales et al. 2023), SNPoptimizer directly optimizes for haplotype uniqueness, quantifying how effectively a candidate SNP combination resolves all genotypes. This objective is conceptually related to the variety discrimination power (VDP), an appraisal index introduced to quantify the ability of a locus combination to distinguish plant varieties (Yang et al. 2021). The algorithm employs iterative selection, crossover, and mutation steps to explore the combinatorial space of markers and converge on near-optimal solutions. The application returns a detailed report including the selected SNPs, the number of unique haplotypes identified, and, if necessary, additional rounds of optimization can be implemented to resolve remaining genotype duplications. We demonstrate that SNPoptimizer directly optimizes multilocus profile resolution and can generate compact, application-specific SNP panels across datasets with different sample sizes and marker densities. Our strategy allows users to generate compact, custom SNP panels suited for routine genotyping assays such as KASP or TaqMan, with direct applicability in both model and non-model plant species.

## Materials and methods

### SNPoptimizer overview

**Fig. 1:**
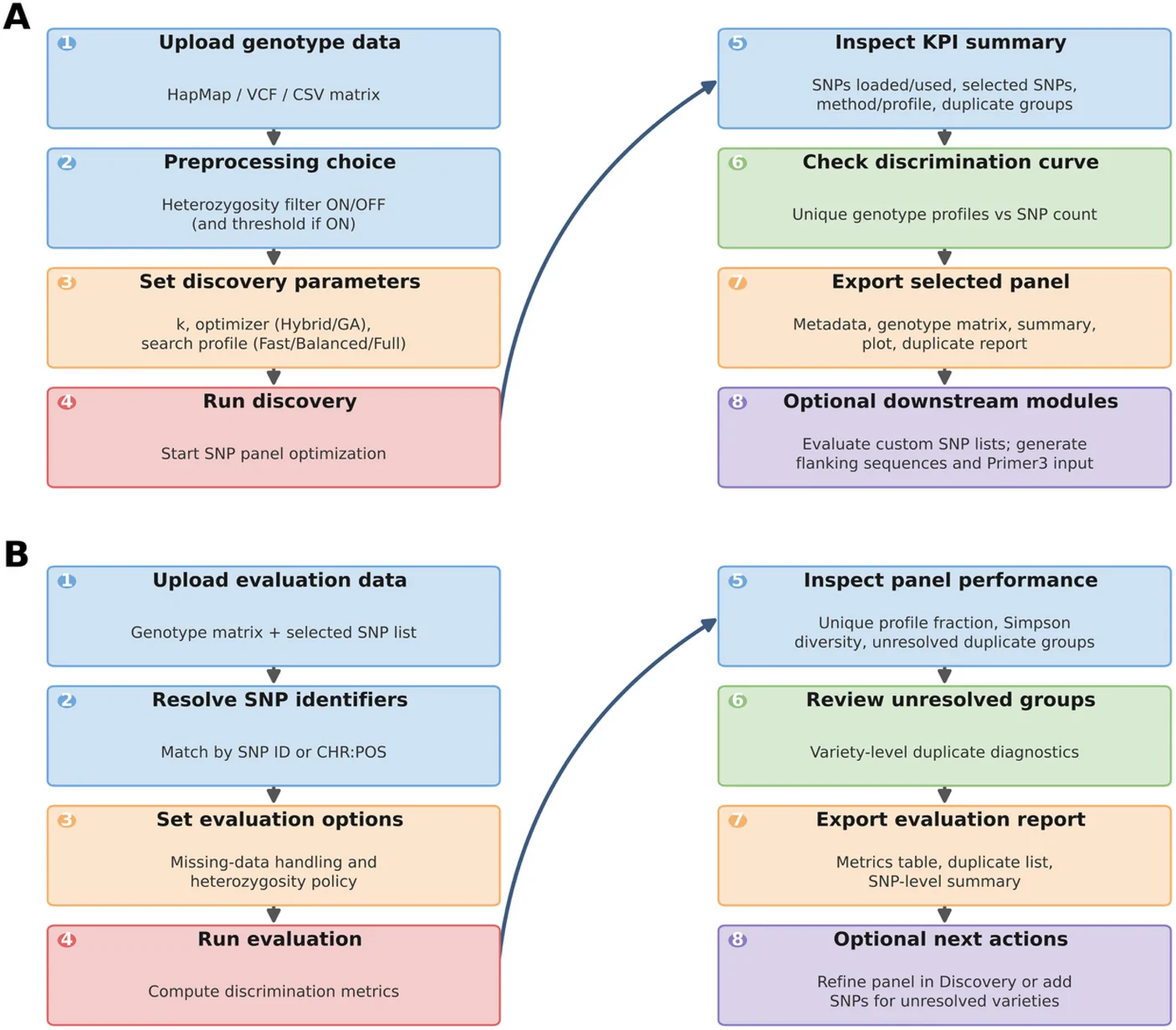
SNPoptimizer graphical interface for panel discovery and panel evaluation. (**A**) Discovery mode. Steps 1–4 include data upload (HapMap/VCF/CSV), optional heterozygosity filtering with a user-defined threshold, definition of optimization settings (k, method: Hybrid or GA, and search profile: Fast/Balanced/Full), and execution of SNP panel discovery. The right side (steps 5–8) reports discovery outputs, including key performance indicators (KPI), discrimination curve (unique profiles as a function of SNP count), downloadable output files (metadata, SNP matrix, summaries, diagnostics), and optional downstream utilities (custom panel evaluation, flanking sequence extraction, Primer3-ready export). (**B**) Evaluation module. Steps 1–4 cover evaluation input and setup: upload SNP data and selected SNP list, marker matching by SNP ID or CHR: POS, and run the evaluation. The right side (steps 5–8) provides panel-quality outputs, including discrimination metrics (unique profile fraction, Simpson diversity, unresolved duplicate groups), duplicate diagnostics, exportable evaluation reports, and optional refinement actions for unresolved varieties

SNPoptimizer was developed entirely in R (R Core Team 2024) and is structured around two components: an initial preprocessing script (main.R), which can be adapted to accommodate new datasets or experimental designs, and a Shiny application (app.R) (Chang et al. 2025), which provides an interactive environment for data exploration, visualization, and rapid hypothesis testing. The preprocessing script manages data import, quality control, genotype encoding, and marker filtering, while the Shiny interface allows users to run the optimization procedure, visualize intermediate results, and directly retrieve the final discriminatory SNP panel. The app supports HapMap, VCF and CSV inputs (IUPAC or GT codes) and offers two complementary workflows: Discovery and Evaluation (Fig. 1). In the Discovery workflow, users begin by uploading a genotype file in HapMap, VCF, CSV formats through the “Upload” section (Fig. 1A). The input file must have SNPs in rows and genotypes in columns, with IUPAC/GT codes indicating homozygous and heterozygous states. No phenotypic data is required. To identify the minimal set of SNPs capable of maximally discriminating all genotypes in a dataset, SNPoptimizer employs two optimization modes: a Genetic Algorithm (GA) (Goldberg 1989; Holland 1992; Haupt 2004; Scrucca 2013, 2017) mode and a Hybrid (greedy + local search) mode. The Evaluation module instead quantifies the discriminative power of an externally defined marker list without running any discovery (Fig. 1B). The module accepts datasets in HapMap, VCF, CSV format, which can be uploaded directly within the panel or reused from the Optimization workflow. Users then provide the target SNPs either by uploading a simple file or by pasting text into a dedicated box. The interface reports the number of requested sites that were successfully matched in the dataset and lists any unmapped positions. From the matched loci, the application extracts the corresponding sub-matrix and computes (i) the total number of unique individuals and the count of residual duplicates and (ii) a discrimination curve showing the cumulative increase in uniqueness as markers are added in the user-supplied order. Also in the Evaluation module, outputs include an on-screen summary (requested vs. matched SNPs, sample count, unique individuals, duplicates), the mapping table, and the discrimination curve.

### SNPoptimizer: discovery, GA, and hybrid optimization framework

SNPoptimizer identifies fixed-size SNP panels for sample discrimination through two optimization engines (GA and Hybrid) embedded in a common discovery workflow (Fig. 1 and Supplementary Fig. 1). Let * m* be the number of candidate SNPs after preprocessing, * N* the number of samples, and * k* the target panel size selected by the user.

#### Discrimination metrics and objective function

Users load SNP data (HapMap, VCF, or CSV), define * k*, and optionally apply a per-locus heterozygosity filter.

For locus * i*, heterozygosity is computed as:

$$\:{h}_{i}=\frac{\#{heterozygous\:calls\:at\:locus\:}i}{\#{non-missing\:calls\:at\:locus\:}i}$$

Loci are retained when:

$$\:{h}_{i}\le\:\tau\:$$

where $\:\tau\:$ is the user-defined threshold (default * 0.05*). If filtering is disabled, all uploaded loci are used unchanged.

SNPs are normalized to symbolic states (A/C/G/T plus IUPAC heterozygous codes), and a numeric matrix is derived in parallel for scoring components.

For any candidate panel * P* with $\:\left|P\right|=k$, SNPoptimizer concatenates the alleles at the k selected loci into a multilocus profile for each sample. Samples sharing an identical profile are assigned to the same profile group *g*, and n_g_ denotes the number of samples (n) assigned to group *g*.

Unresolved sample pairs are computed as:

$$\:U\left(P\right)=\sum\:_{g}\frac{{n}_{g}\left({n}_{g}-1\right)}{2}$$

Total sample pairs are:

$$\:T=\frac{N\left(N-1\right)}{2}$$

Pairwise discrimination is:

$$\:{D}_{\mathrm{pair}}\left(P\right)=1-\frac{U\left(P\right)}{T}$$

Unique-profile fraction is:

$$\:{U}_{\mathrm{frac}}\left(P\right)=\frac{G\left(P\right)}{N}$$

where $\:G\left(P\right)$ is the number of distinct multilocus profiles and *N* is the total number of samples. Alleles across the *k* selected loci are concatenated into a multilocus profile.

Simpson’s diversity is:

$$\:S\left(P\right)=1-\frac{\sum\:_{g}{n}_{g}\left({n}_{g}-1\right)}{N\left(N-1\right)}$$

Missingness is measured as:

$$\:M\left(P\right)=\mathrm{mean\:sample-wise\:missing\:call\:proportion\:across\:selected\:SNPs}$$

During optimization, candidate panels are ranked using a discrimination-prioritized search score:

$$\:{F}_{\mathrm{search}}\left(P\right)={D}_{\mathrm{pair}}\left(P\right)+0.08\hspace{0.17em}{U}_{\mathrm{frac}}\left(P\right)+0.02\hspace{0.17em}S\left(P\right)-0.01\hspace{0.17em}M\left(P\right)$$

Panel comparison is lexicographic: (i) fewer unresolved pairs, (ii) fewer duplicated samples, (iii) more unique profiles, (iv) higher $\:{F}_{\mathrm{search}}$. The numerical coefficients in $\:{F}_{\mathrm{search}}\:$are fixed heuristic weights used to guide the search and were not empirically fitted to the benchmark datasets. These coefficients do not by themselves enforce the priority hierarchy. Final candidate panels are compared lexicographically according to the number of unresolved pairs, duplicated samples, and distinct profiles, with $\:{F}_{\mathrm{search}}$ used as the final tiebreaker.

#### GA mode

In GA mode, each candidate panel is represented as a binary chromosome of fixed cardinality * k* over a reduced candidate pool. A deterministic repair operator enforces:

$$\:\sum\:_{j=1}^{m{\prime\:}}{b}_{j}=k$$

where $\:m{\prime\:}$ is the reduced pool size and $\:{b}_{j}\in\:\{0,1\}$.

The GA applies selection, crossover, mutation, and elitism across generations. Initialization is seed-informed (greedy/beam-derived seeds plus rank-based seeds), not purely random, to improve convergence stability. Multiple restarts are executed with controlled exploration-exploitation balance; each run is followed by local post-GA refinement (single-marker swaps and duplicate-focused swaps). Optimization stops at convergence or maximum iterations.

#### Hybrid mode

In Hybrid mode, SNPoptimizer first ranks informative candidates and constructs an initial * k*-SNP panel by greedy forward selection (largest immediate gain in discrimination). This panel is then improved through local search and targeted SNP swaps. The Hybrid strategy includes (i) multi-start generation of restart panels from partially randomized high-ranking pools; (ii) duplicate-focused refinement on unresolved groups, and (iii) Optional intensified tail refinements (targeted * k*-opt and last-mile shaking) when unresolved pairs are few. This design preserves high discrimination while reducing runtime versus deep GA-only searches in large marker spaces.

#### Additional duplicate-resolution run

If unresolved duplicate groups remain, SNPoptimizer can perform a second discovery step on duplicate samples only, selecting $\:{k}_{\mathrm{add}}$ additional loci from the remaining candidate set. This optional stage is intended for practical workflows where users prefer adding few markers instead of replacing the whole panel.

#### Search profiles and runtime-depth trade-off

Search depth is user-configurable:

- Fast: reduced runs/restarts and lighter refinement (shortest runtime).
- Balanced: default compromise between speed and search depth.
- Full: expanded runs/restarts and deeper refinement caps (highest search depth, longest runtime).

Runtime is mainly driven by sample size (* N*), number of scoring evaluations, and selected profile.

#### Reporting score

For result reporting, SNPoptimizer also computes a summary score integrating discrimination, diversity, missingness, and redundancy:

$$\:{F}_{\mathrm{report}}\left(P\right)=0.6\hspace{0.17em}{U}_{\mathrm{frac}}\left(P\right)+0.25\hspace{0.17em}S\left(P\right)-0.1\hspace{0.17em}M\left(P\right)-0.05\hspace{0.17em}LD\left(P\right)$$

where $\:LD\left(P\right)$ is the mean absolute pairwise correlation among selected loci (numeric encoding). In contrast to F_search_(P), the reporting score F_report_(P) is a summary computed for the final panel; its coefficients (0.6, 0.25, 0.1, 0.05) weight discrimination, diversity, missingness and redundancy on a common 0–1 scale to give users an interpretable single-number description of panel quality. The two functions therefore serve distinct and non-redundant purposes: F_search_(P) is the optimisation objective (discrimination-first, with weak tiebreakers), whereas F_report_(P) is a balanced post-hoc descriptor that also penalises marker redundancy and missing data, which are not part of the optimisation objective.

### Example data

To evaluate the performance and flexibility of SNPoptimizer, three genotypic datasets representing diverse species, panel sizes, and SNP densities were used.

- Dataset I – Tomato, Varitome Project (Mata-Nicolás et al. 2020).

The dataset consisted of 166 tomato accessions from the Varitome collection, which captures the continuum from wild (*S. pimpinellifolium*) to semi-domesticated (*S. lycopersicum* var. cerasiforme) and cultivated (*S. lycopersicum* var. lycopersicum) tomatoes (Mata-Nicolás et al. 2020). Genotypic data were derived from whole-genome resequencing, and variant calling results in VCF format were downloaded from SolGenomics (https://solgenomics.net/). Raw data (PRJNA454805) were filtered with plink (Purcell et al. 2007) to remove all heterozygous and missing loci, resulting in a final dataset of ~ 350,000 high-quality SNPs.

- Dataset II – Cauliflower (Yang et al. 2022)

The dataset was published by Yang et al. (2022). The variant calling data (1,662 high-quality biallelic SNPs) of 820 cauliflower inbred lines sequenced with the NovaSeq 6000 Sequencing System (Illumina, USA) were downloaded from Zenodo (accession: 7179139).

- Dataset III – Public soybean whole-genome diversity panel (Zhang et al. 2022).

Dataset III was derived from the public 1.5 K soybean whole-genome resource described by Zhang et al. (2022) and deposited in the USDA database (https://doi.org/10.15482/USDA.ADC/1519167). The chromosome-level VCF contained 30,919,090 variants for 1,511 samples. Samples with more than 20% missing genotypes were removed, whereas heterozygous outliers were flagged but not excluded. Markers were then filtered based on minor-allele frequency > = 0.01, call rate > = 0.90, and observed heterozygosity < = 0.05, yielding the final matrix with 1,499 samples and 1,002,178 SNPs. Dataset III also served to test computational performance. Reproducible subsets of 100,000, 250,000, 500,000, and 1,000,000 SNPs were tested with the number of samples fixed at 100, whereas subsets of 100, 250, 500, 1,000, and 1,499 accessions were evaluated with the number of markers held fixed at 100,000.

### Assessment of discrimination power (R-VDP)

To enable direct, coefficient-independent comparison across tools and datasets, here we additionally reported the resolving variety discrimination power (R-VDP) (Yang et al. 2021). For cross-tool evaluation, ratio-based variety discrimination power (R-VDP) was calculated following Yang et al. (2021) as the number of distinct multilocus profile groups divided by the total number of samples. A perfect-match threshold was used, whereby two samples were considered different when they differed at one or more selected loci.

## Results and discussion

### Application to trial datasets

The Discovery mode was applied to three datasets to benchmark the tool across different genomic contexts. The three empirical datasets span complementary regimes rather than a biological comparison. Indeed, tomato represents a dense resequencing panel across *S. lycopersicum* domestication, cauliflower represents a curated low-density fingerprinting panel, and soybean represents an agronomically relevant diversity collection containing cultivated and wild accessions. Since the datasets differ in sample size, markers, missingness, and population composition, all factors that may affect both runtime and the number of resolvable profiles, panel sizes and discrimination rates were interpreted within each dataset rather than compared directly across them. For each dataset, the number of selected SNPs, duplicates resolved, and performance across GA iterations were recorded and compared. The parameters of the genetic algorithm (population size and number of iterations) were selected to balance computational efficiency and optimization performance. In SNPoptimizer, the population size determines the number of candidate SNP subsets explored at each generation, while the iteration number controls the depth of the search across the solution space. We adopted values commonly used in GA-based feature selection problems, where moderate population sizes ensure sufficient genetic diversity among solutions, and a limited number of iterations prevents premature convergence while keeping runtime manageable. The genetic algorithm parameters (population size and number of iterations) were empirically optimized to achieve the best overall performance across datasets. We conducted a series of exploratory runs in which these parameters were varied systematically, and we selected the combination that consistently produced the highest discriminatory power with stable convergence and acceptable computational time.

Dataset I originates from the Varitome project: “Exploitation of genetic and epigenetic variation in the regulation of tomato fruit quality traits”, available at SolGenomics (https://solgenomics.net) (Mata-Nicolás et al. 2020). The Varitome collection comprises a genetically diverse tomato panel that spans the continuum from wild species (*S. pimpinellifolium*) to semi-domesticated populations (*S. lycopersicum* var. *cerasiforme*) and ancestral landraces (*S. lycopersicum* var. *lycopersicum*). It reflects the evolutionary trajectory from wild to cultivated tomato and was designed to capture genetic variants and alleles lost during domestication. For this subset, a total of 166 tomatoes and ~ 350,000 filtered variants were used. Using SNPoptimizer and hybrid mode, a first-round of 20 SNPs successfully resolved 156 out of 166 samples (Table 1). A second-round analysis, with two additional SNPs, did not improve the results, suggesting the presence of putative redundant profiles (Table 1). For this subset, eight duplicated groups were identified.

**Table 1:** Benchmark comparison of SNPoptimizer versus minSNP and LociScan. The number of distinct discriminated genotypes over the dataset size, running time (s) and R-VDP scores were also reported

Tool	Dataset	# samples	# markers	Running Time	Distinct Genotypes	*R*-VDP
SNPoptimizer	Dataset I	166	409,136	≈ 110 s	156	0.94
	Dataset II	820	1,662	≈ 4 s	717	0.87
	Dataset III	1,499	100,000	406.15 s	1,495	0.99
minSNP	Dataset I	166	409,136	> 10 h	160	0.96
	Dataset II	820	1,662	113.29 s	738	0.90
	Dataset III	1,499	100,000	13,430.20 s	1,499	1.00
LociScan	Dataset I	166	409,136	1.94 s	147	0.89
	Dataset II	820	1,662	23.94 s	689	0.84
	Dataset III	1,499	100,000	144.14 s	1,400	0.93

Although this step targeted only a small group of individuals, the additional markers captured subtle allelic differences that were not represented in the first-round solution, attempting to discriminate the most genetically similar individuals. Overall, SNPoptimizer achieved a discrimination accuracy of 94.0% (computed as the number of uniquely resolved samples divided by the total number of samples, e.g., 156/166) in the dataset, characterized by close genetic relationships and overlapping evolutionary histories.

**Fig. 2:**
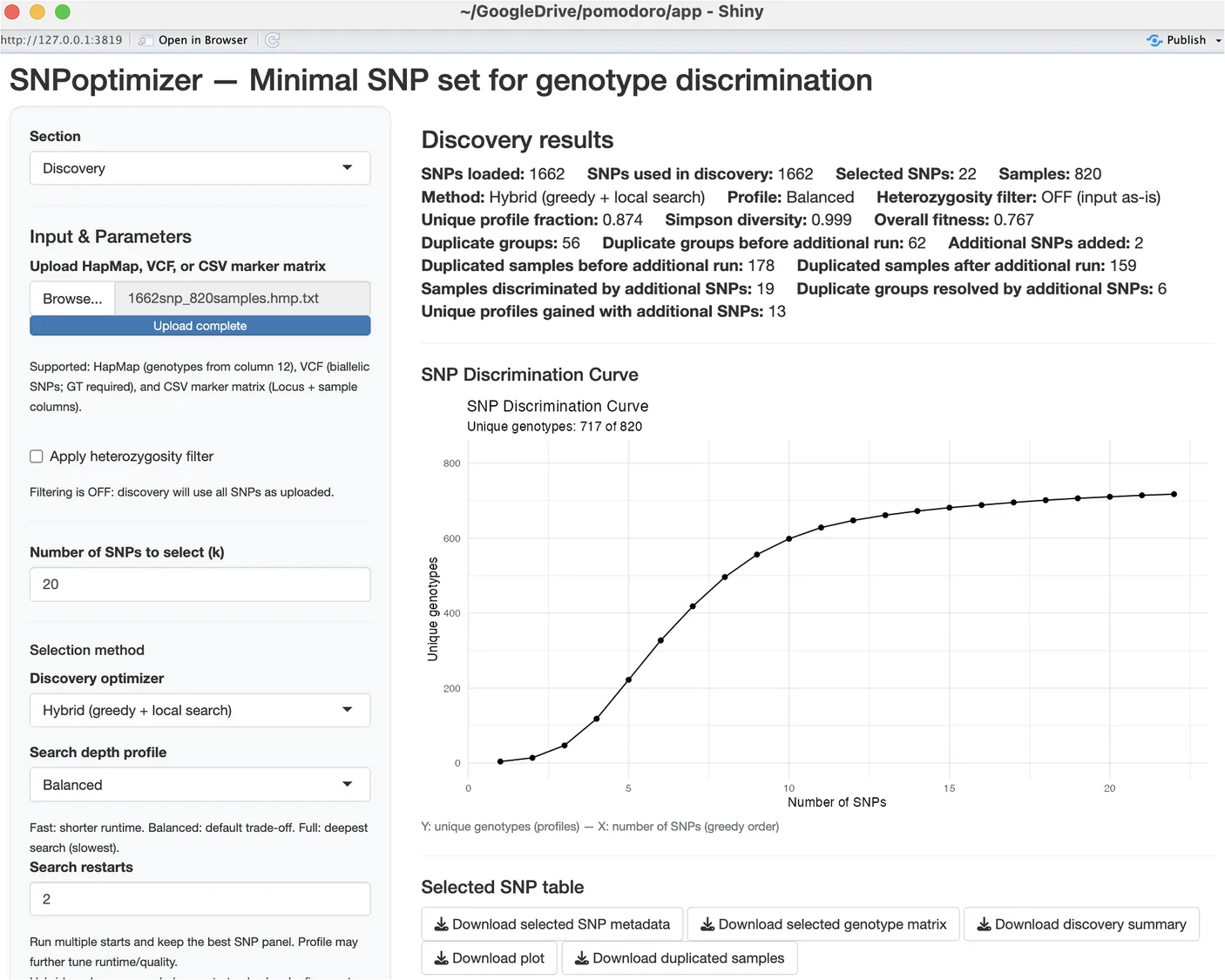
Discovery interface and performance output. Screenshot of the SNPoptimizer Shiny application showing the Discovery results panel. The interface summarizes the input dataset characteristics (number of SNPs, samples, and detected duplicates), the selected optimization parameters, and the SNP discrimination curve. The curve illustrates the relationship between the number of selected SNPs and the proportion of uniquely discriminated samples, highlighting the plateau where additional SNPs provide diminishing returns. The panel also reports the number of duplicated samples resolved and provides downloadable outputs, including the selected SNP list and summary statistics

Dataset II derives from Yang et al. (2022), who established a SNP-based fingerprinting system for cauliflower (*Brassica oleracea* var. *botrytis*) using the KASP genotyping platform. In their study, 820 inbred lines were resequenced, and ~ 17.9 million SNPs were identified through a GATK-based variant calling pipeline. A stringent multi-step filtering process led to the selection of 1,662 high-quality SNPs, chosen for high polymorphism, low heterozygosity, and absence of flanking polymorphisms (Yang et al. 2022). These markers were subsequently used for downstream fingerprinting and population analysis. However, the final panel of 41 KASP SNPs used for varietal identification was selected through *in silico* simulations conducted on a reduced core set of 153 representative accessions. The selected SNPs were then validated on 329 commercial cultivars, enabling their classification into three major genetic clusters based on curd solidity and geographical origin. In contrast, we applied SNPoptimizer directly to the complete set of 820 inbred lines, using the full matrix of 1,662 SNPs as input (Fig. 2). In the first discovery round with hybrid mode, a subset of 20 SNPs was selected, which uniquely resolved 642 samples (Table 1). To address the remaining unresolved duplicates, a second discovery round was performed, selecting 2 additional SNPs. This brought the total panel to 22 SNPs, enabling the discrimination of 717 unique individuals (Table 1, Fig. 2). The SNP discrimination curve generated by SNPoptimizer shows a steep resolution increase in the first 10–15 SNPs, followed by a plateau phase, highlighting the genetic redundancy within the full dataset. The result demonstrated that, even when no prior reduction to a core set is performed, SNPoptimizer can identify compact SNP subsets that maximize resolution in large and complex populations. Unlike predefined simulation-based approaches, this method adapts dynamically to the structure of the full matrix and allows users to tailor the discovery to specific panel sizes and resolution goals.

For Dataset III, SNPoptimizer was applied to 1,499 retained soybean accessions. Using the Hybrid method with k = 17, population size 30, 40 iterations, one restart, and one ensemble run, the nested 100,000-SNP comparison panel produced a median of 1,495 distinct profiles across three seeded runs (range 1,495–1,499; median R-VDP = 0.99) in a median search time of 406.15 s (range 389.91–406.50 s). Two runs left four profiles unresolved, whereas one achieved complete discrimination (Table 1). Repeating the benchmark once with all 1,002,178 post-QC candidate SNPs produced 1,495 distinct profiles (R-VDP = 0.99), indicating no observed discrimination gains relative to the three-run median at k = 17, while search time increased to 3,753.06 s. The all-candidate endpoint is a single seeded stress test and is not an estimate of run-to-run variability.

### Computing time and resource usage

To assess computational performance, all benchmarks were run on traceable hardware, with the search configuration reported alongside each result. The original Varitome tomato analysis (~ 350,000 SNPs, 166 samples; PopSize = 50, 900 iterations, 20 + 2 SNPs) required ~ 110 s, and the curated cauliflower analysis (1,662 SNPs, 820 samples; PopSize = 150, 900 iterations, 20 + 2 SNPs) ~ 4 s, both on an Apple M1 Pro workstation with 32 GB RAM. Since the two datasets differ in sample number, marker number, and search settings, their absolute times cannot isolate a single scaling factor. We therefore used Dataset III as a controlled stress test. On all 1,499 accessions, expanding the marker pool from 100,000 to 1,002,178 SNPs increased median search time from 406.15 to 3,753.06 s with a 9.24-fold rise for a 10.02-fold increase in markers. Total R elapsed time was 3,770.59 s and peak vector-heap use ~ 17.8 GB, demonstrating feasibility for ~ 10⁶ post-QC SNPs and ~ 1,500 samples on a 32-GB workstation. In addition, to separate the two dimensions (samples and markers), we then varied one factor at a time on Dataset III, using identical hardware and Hybrid settings. Along the marker axis, exactly nested panels of 100,000–1,000,000 SNPs were evaluated with samples fixed at 100; along the sample axis, stratified nested subsets of 100–1,499 accessions were evaluated with markers fixed at 100,000 (Supplementary Fig. 2). Median search time varied from 76.42 to 729.63 s across the marker axis, giving a log–log slope of 0.981 (HC3 95% CI 0.897–1.066; ×1.97 per doubling; R² = 0.9998). At 1,499 samples, two of three runs resolved 1,495 profiles and one resolved all, so convergence and matrix composition also shape elapsed time. The sample-number slope was therefore uncertain (0.854; HC3 95% CI − 0.634–2.342; R² = 0.721) and did not differ significantly from the marker-axis slope (interaction *p* = 0.797). These results support near-linear runtime growth with candidate-SNP number in the tested regime but do not support that marker number is a stronger general driver than sample number. Indeed, tens of thousands of samples remain untested. Mechanistically, genome-wide candidate ranking scales linearly with both the number of samples (N) and the number of candidate SNPs (m), since it inspects genotypes across all loci and samples; evaluating a single restricted k-locus panel instead scales linearly with N and the fixed panel size k.

### Comparative evaluation of SNPoptimizer with existing tools

Several approaches have been proposed to maximize genetic diversity in germplasm collections, particularly for core collection development (Gu et al. 2023; Yang et al. 2024; Gouy et al. 2025; Zhao et al. 2025). However, relatively few tools are specifically designed to identify a minimal subset of SNP markers that uniquely discriminates all samples in a dataset, offering a user-friendly interface with minimal parameter settings. In many cases, the procedure is implemented ad hoc, with short scripts embedded in the methods section or supplementary material, rather than being released as reusable software. For example, in Dataset II (cauliflower), Yang et al. (2022) performed in silico simulations to select 41 SNPs for varietal identification from an initial set of 1,662 high-quality markers. This was achieved through a Python-based program that selects the most suitable set of SNPs by in silico simulation on (https://github.com/Lvmingjie/SNP-fingerprints-of-Cauliflower, CoreSNPSimulation.py). However, the absence of details regarding the input format files makes the program difficult to compare this dataset with the other datasets used in this study. SNPoptimizer enabled the discrimination of 717 unique samples (Dataset II), which might offer superior discriminating power compared to the 41 KASP markers developed and validated on 329 commercial cultivars by Yang et al. (2022). In contrast, minSNPs is a fully implemented and publicly available R package designed to derive resolution‑optimized SNP sets from genomic data (Hoon et al. 2023). It accepts aligned sequences in FASTA format or genotype matrices encoded with IUPAC nucleotide codes, and provides metrics for SNP selection, including the Simpson index, which maximizes overall sample diversity (Simpson 1949). The tool can identify SNP panels of user‑defined size, evaluate their discriminatory power, and optionally optimize for specific groups of interest. Here, we applied minSNPs to example datasets. This enables a direct comparison of resolution power, number of unique individuals identified, and computational efficiency. While minSNPs successfully identified 22 SNPs capable of resolving 160 unique genotypes (leaving 6 duplicates) after more than 10 h of computation, SNPoptimizer resolved 156 unique genotypes in less than 5 min on an Apple M1 Pro, 32 GB RAM, using a population size of 20 and 900 iterations. On Dataset II, minSNPs resolved slightly more profiles than SNPoptimizer (738 vs. 717; Table 1). However, SNPoptimizer required approximately 4 s, whereas minSNPs required a median of 113.29 s (range: 113.20–114.05 s). For soybean Dataset III, all three tools received the identical 100,000-SNP × 1,499-sample matrix. Across three seeded SNPoptimizer runs at k = 17, the median result was 1,495 profiles (range 1,495–1,499; median R-VDP = 0.9973) in 406.15 s (range 389.91–406.50 s). A complete minSNPs search with maximum depth 17 reached full discrimination at k = 16 (1,499 profiles; R-VDP = 1.00) in 13,430.20 s. Across three LociScan runs at k = 17, the median result was 1,400 profiles (range 1,397–1,436; median R-VDP = 0.9340) in 144.14 s (range 127.94–185.17 s). Thus, LociScan was fastest under these settings, minSNPs achieved the highest discrimination with one fewer marker but required substantially longer, and SNPoptimizer provided near-complete discrimination with an intermediate runtime.

In addition, we evaluated SNPoptimizer on the same benchmark datasets used by LociScan (Yang et al. 2024). On the rice dataset (387 varieties), LociScan reported full discrimination at (k = 17). In our runs, SNPoptimizer reproduced this threshold and also identified fully discriminative panels at (k = 16) in a subset of runs (Table 2). At (k = 16), Hybrid outperformed GA in both robustness and runtime: full discrimination was achieved in 7/30 runs (23.3%) for Hybrid versus 4/30 runs (13.3%) for GA. Median of unique varieties resolved was 368/387 for Hybrid and 366/387 for GA, with a higher median score for Hybrid (0.814 vs. 0.811). Runtime distributions were right-skewed for both methods, but Hybrid remained faster in typical runs (median 225 s vs. 272 s) and showed a much lower best full-solution time (271 s vs. 1286 s). At (k = 17), Hybrid was fully stable (30/30 full runs; 387/387), with a median score of 0.844 (range 0.842–0.845) and median runtime 319 s (min 137 s; p90 764 s), while GA reached 29/30 full runs. Overall, SNPoptimizer matches the LociScan (k = 17) target on rice and additionally shows the potential to reach full discrimination with fewer markers (k = 16) in part of the runs. Across the maize benchmark (93 varieties), LociScan reports a best panel size of (k = 7). SNPoptimizer reproduced this threshold: at (k = 7), Hybrid achieved full discrimination in 30/30 runs (100%), while GA achieved 29/30 runs (96.7%). As expected, neither method reached full discrimination at (k = 5) or (k = 6) (0/30 for both), indicating that full variety resolution in this dataset is strongly constrained below 7 loci. At suboptimal (k), Hybrid showed faster median runtime than GA (e.g., 34.9 s vs. 40.2 s at (k = 5); 27.7 s vs. 32.9 s at (k = 6) with comparable discrimination levels.

Using R-VDP as the unified coefficient-independent metric, SNPoptimizer reached median R-VDP values of 1 on rice at k = 17 and maize at k = 7 (Table 2). In 30 independent runs at the same panel sizes, LociScan produced median R-VDP values of 0.9535 for rice and 0.9570 for maize, with no fully discriminating run; median program-reported times were 6.46 s and 0.40 s, respectively (Table 2). These results showed that, under the tested settings, SNPoptimizer achieved higher discrimination at the same k.

**Table 2:** Unified comparison of SNPoptimizer and LociScan on rice and maize datasets. SNPoptimizer values are medians across 30 independent runs per method and panel size. Full runs and full rate quantify complete discrimination

Dataset	Method	k	Runs	Full runs	Full rate (%)	Median *R*-VDP	Median time (s)
Rice	GA	16	30	4	13.3	0.94	272.3
Rice	Hybrid	16	30	7	23.3	0.95	225.4
Rice	GA	17	30	29	96.7	1.00	376.3
Rice	Hybrid	17	30	30	100	1.00	319.3
Rice	LociScan	17	30	0	0	0.95	6.46
Maize	GA	6	30	0	0	0.83	32.9
Maize	Hybrid	6	30	0	0	0.84	27.7
Maize	GA	7	30	29	96.7	1.00	122
Maize	Hybrid	7	30	30	100	1.00	160.7
Maize	LociScan	7	30	0	0	0.95	0.40

It is noteworthy that the aim of the comparison is not only to demonstrate that SNPoptimizer outperforms all existing tools, but rather to evaluate its performance in terms of computational efficiency, scalability, and discriminative power under comparable conditions. Given the diversity of available approaches for SNP selection, each relying on different optimization strategies and objectives, performance should be interpreted within the specific context of the datasets and evaluation criteria considered. The differences observed between GA and the hybrid optimization strategy implemented in SNPoptimizer suggest that the latter approach can improve solution quality under specific conditions. While the GA efficiently explores the combinatorial search space and rapidly identifies near-optimal SNP subsets, it may converge prematurely to fully refine solutions in datasets with complex structure. The hybrid approach, which integrates the exploratory power of the GA with additional refinement or deterministic selection steps, appears to enhance discrimination by improving local optimization of candidate SNP panels. This may explain why the hybrid strategy consistently outperforms the GA alone in certain datasets, where fine-scale resolution among closely related genotypes is required. These results indicate that combining global stochastic search with local optimization can provide a more balanced trade-off between exploration and exploitation, ultimately leading to improved marker selection performance. More broadly, these results highlight how combining global stochastic search with local optimization can provide a more effective balance between exploration and exploitation, improving both solution quality and robustness. At the same time, the GA-based framework of SNPoptimizer enables rapid convergence and scalability to large datasets, supporting its applicability in high-dimensional marker selection problems.

Overall, these results indicate that Hybrid is the preferred default engine for varietal discrimination tasks: compared with GA, it provides higher full-resolution success at lower cardinalities, better median discrimination metrics, and better time-to-solution for successful panels.

### Pre-processing considerations

Appropriate pre-processing of the input SNP matrix is essential to ensure that marker selection is driven by true genetic differences rather than by technical artefacts, inconsistent genotype coding, or missing data patterns. Before running SNPoptimizer, users should retain only high-quality biallelic SNPs, remove markers with excessive missing data, and exclude samples with high genotype missingness. This step is particularly important because missing calls can artificially inflate apparent genotype uniqueness and may lead the optimization procedure to select markers that distinguish samples because of incomplete data rather than genuine allelic variation. A MAF filter should also be applied before optimization. Near-monomorphic loci generally contribute little to genotype discrimination while increasing the search space and computational burden. As a general starting point, markers with more than 10–20% missing calls and samples with more than 20% missing data can be removed, while a MAF threshold in the range of 0.01–0.05 is suitable for many diversity panels. These thresholds should not be interpreted as fixed requirements, because optimal filtering depends on the species, ploidy level, reproductive biology, genotyping platform, marker density, and the intended application of the SNP panel. The treatment of heterozygous calls requires particular attention. For predominantly self-pollinating species, highly inbred lines, doubled haploids, or fixed breeding material, unexpectedly high heterozygosity at individual loci may indicate residual genotyping errors, paralogous alignments, poor marker specificity, or sample contamination. In these cases, the heterozygosity filter implemented in SNPoptimizer can be used to remove loci exceeding the selected heterozygosity threshold. Conversely, in outcrossing species, clonally propagated crops, highly heterozygous germplasm, or animal populations, heterozygosity is often biologically informative and should not be treated as an artefact. In such cases, the heterozygosity filter should be relaxed or disabled to avoid removing informative markers. Before upload, users should verify that genotype encoding is consistent across loci and samples, that missing values are coded uniformly, and that sample identifiers are unique, stable, and free of formatting inconsistencies. Duplicate sample names, mixed allele encodings, inconsistent separators, or hidden spaces in identifiers can compromise downstream genotype discrimination and should be corrected during data preparation. For the optimization step, PopSize values between 50 and 150 and approximately 900 iterations provide a practical initial configuration for many datasets. Larger population sizes and additional iterations may be required for very dense SNP datasets, highly redundant marker pools, large germplasm collections, or cases in which repeated runs do not converge toward stable marker subsets. In the datasets analysed in this study, these settings generally provided full or near-full genotype discrimination; however, they should be regarded as recommended starting values rather than universal defaults. Users are encouraged to repeat the optimization with different random seeds or parameter settings to assess the stability of the selected SNP panel before final deployment.

### Broader applications, and limitations

SNPoptimizer was designed to support the selection of minimal highly informative SNP subsets for genotype discrimination. By reducing large marker datasets to compact panels of non-redundant and highly discriminant loci, the tool facilitates the development of cost-effective genotyping assays while preserving the resolution required to distinguish closely related accessions, cultivars, breeding lines, or individuals. This makes SNPoptimizer particularly useful in contexts where large-scale genotyping is impractical or unnecessarily expensive, but robust identification remains essential. Potential applications include varietal registration and protection, DNA fingerprinting, seed purity and lot identity verification, Genebank curation, duplicate detection in germplasm collections, and traceability of plant genetic resources (PGR) along conservation, breeding, and seed supply chains. Although developed with plant genomic resources in mind, the same optimization framework can be extended to other biological systems, including animal genomic selection, parentage verification, breed traceability, and the design of targeted SNP panels for routine molecular certification.

Importantly, the expected performance and optimal marker configuration may differ depending on the specific application. For example, varietal registration typically requires clear discrimination among closely related elite cultivars, whereas genebank curation may involve highly diverse accessions spanning broad geographic or evolutionary backgrounds. In the latter case, fewer markers may be sufficient to separate highly divergent genotypes, while distinguishing among closely related breeding lines often requires more informative loci targeting subtle allelic differences. The effectiveness of SNPoptimizer is highly dependent on the diversity, representativeness, and structure of the input dataset. In panels characterized by high genetic redundancy (e.g., closely related breeding materials), the algorithm may require a larger number of SNPs to achieve full discrimination. Conversely, in highly diverse germplasm collections, minimal marker sets may achieve complete resolution with fewer loci. Therefore, the diversity spectrum of the input genotypes directly influences the size and composition of the resulting SNP panel. The origin and nature of genotyping data also influence performance. SNPs derived from whole-genome sequencing (WGS) datasets may provide a more uniform genome-wide representation but can include uneven coverage and higher missingness rates. In contrast, SNP arrays or pre-filtered genotyping platforms often introduce ascertainment bias, as markers are selected based on prior polymorphism discovery panels. Such biases can affect allele frequency distribution and the informativeness of loci in external populations. Users should therefore consider the underlying marker discovery strategy when interpreting panel transferability and resolution power. The treatment of heterozygous calls represents another critical factor. In predominantly self-pollinating species or advanced inbred lines, high heterozygosity may reflect genotyping errors or residual heterogeneity and may require stringent filtering. In contrast, in outcrossing species or clonally propagated crops, heterozygosity is biologically meaningful and contributes to discriminatory power. SNPoptimizer allows adjustable filtering thresholds to accommodate these biological differences. Finally, the reliability of the output matrix and the discriminatory performance of the selected SNP panel depend strongly on data consistency, including genotype encoding schemes, missing data thresholds, allele recoding, and uniform sample naming. Proper pre-processing and quality control remain essential prerequisites.

When applied to well-curated, representative, and high-quality genotype datasets, SNPoptimizer provides a robust and flexible framework for reducing large-scale SNP matrices into actionable, compact marker panels tailored to specific objectives, from PGR conservation and variety distinction to breeding support and traceability systems.

## Conclusions

SNPoptimizer provides a powerful, intuitive, and computationally efficient solution for researchers and breeders seeking to streamline genotyping workflows without sacrificing resolution. Its scalability to large SNP datasets and flexibility across different genotyping platforms (e.g., sequencing- and array-based data) further enhance its applicability in diverse research and breeding scenarios. By enabling the identification of highly informative SNP subsets from large datasets, the tool bridges the gap between high-throughput sequencing data and practical downstream applications such as varietal registration, germplasm documentation, and seed traceability. Its flexible design, combined with biologically meaningful filtering options and interactive visualizations, makes SNPoptimizer suitable for a wide range of species, experimental contexts, and user expertise levels. Although demonstrated here on plant datasets, the underlying approach is generalizable and could be extended to other domains, including microbial typing, livestock genomics, and population genetics studies. As global efforts in plant breeding and biodiversity conservation increasingly rely on molecular data, tools like SNPoptimizer are essential for transforming complex genomic information into clear, cost-effective, and actionable outputs. Whether applied to core collection fingerprinting, seed certification, or germplasm management, SNPoptimizer enables users to translate genomic data into rapid and reliable decisions.

## Supplementary Information

Below is the link to the electronic supplementary material.

Supplementary Material 1 (DOCX 361 KB)

Supplementary Material 2 (DOCX 236 KB)

## Data Availability

No datasets were generated or analysed during the current study.

## Declarations

**Project name**: SNPoptimizer.

**Project home page**: https://github.com/salvatoreesposito01/SNPOpt.

**Operating system(s)**: Platform independent.

**Programming language**: R (version ≥ 4.2).

**Other requirements**: R packages: shiny (Chang et al. 2025), GA (Scrucca 2013; 2017), data.table (Barrett et al. 2026), tidyverse (Wickham et al. 2019), DT (Xie et al. 2025), ggplot2 (Wickham 2016), plotly (Sievert 2020), shinyWidgets (Perrier et al. 2026), BiocManager (Morgan et al. 2025), Biostrings (Pagès et al. 2025), parallel (Corporation et al. 2022), shinycssloaders (Attali et al. 2025).

**Browser**: Any web browser (Chrome, Firefox, Safari, Edge).

**License**: GPL-3.

**Any restrictions to use by non-academics**: No restrictions for GPL-3 (commercial use allowed under license terms).

### Generative AI and AI-assisted technologies

During the preparation of this work, the authors used two generative-AI tools: Codex 5.3-codex (OpenAI; accessed up to January 2026) and ChatGPT (OpenAI, GPT5.5; accessed up to March 2026). These tools assisted with (i) revising and debugging Python scripts ii) improving the text to improve clarity and readability. All AI-generated suggestions were critically reviewed and edited by the authors. The authors take full responsibility for the integrity and accuracy of all content presented in this manuscript.
