# Supplementary material for: SNPoptimizer: a scalable genetic-algorithm framework to derive minimal discriminatory SNP panels from large genotyping datasets
Source: Mol Breed. 2026 Aug 29;46(9):89. doi: 10.1007/s11032-026-01707-z (PMC13525983; doi:10.1007/s11032-026-01707-z)
Supplement: Supplementary file 1 [file 11032_2026_1707_MOESM1_ESM.docx]

**Supplementary Figure 1.** **Optimization strategies for SNP panel discovery.** (A) GA strategy: a population of (k)-SNP candidate panels is initialized and iteratively updated through selection, crossover, and mutation. Candidate panels are scored by variety discrimination, and evolution proceeds until convergence or the maximum number of iterations is reached, followed by local/duplicate-focused refinement of the best panel. (B) Hybrid strategy: informative SNPs are first ranked to construct an initial (k)-SNP panel by greedy search. Multiple restart panels are then explored and refined through SNP-swap local improvement and duplicate-focused optimization. When only a few unresolved pairs remain, a last-mile targeted refinement is applied before outputting the final panel.
