# Supplementary material for: SNPoptimizer: a scalable genetic-algorithm framework to derive minimal discriminatory SNP panels from large genotyping datasets
Source: Mol Breed. 2026 Aug 29;46(9):89. doi: 10.1007/s11032-026-01707-z (PMC13525983; doi:10.1007/s11032-026-01707-z)
Supplement: Supplementary file 2 [file 11032_2026_1707_MOESM2_ESM.docx]

**Supplementary Figure 2.** Runtime scaling of SNPoptimizer on Dataset III (Hybrid search, k = 17). Search time (seconds; log-scaled) was plotted against candidate-SNP number (left; 100 samples fixed) and sample number (right; the same 100,000 SNPs fixed). Panels were exactly nested, and three runs per condition were conducted using identical hardware and software. Grey points and lines show individual runs; red points and lines show condition medians. Slopes and HC3 95% confidence intervals were from log–log models fitted to condition medians.
